# The impact of the three degrees‐of‐freedom fiducial marker‐based setup compared to soft tissue‐based setup in hypofractionated intensity‐modulated radiotherapy for prostate cancer

**DOI:** 10.1002/acm2.12603

**Published:** 2019-05-04

**Authors:** Satoshi Tanabe, Satoru Utsunomiya, Eisuke Abe, Hiraku Sato, Atsushi Ohta, Hironori Sakai, Takumi Yamada, Motoki Kaidu, Hidefumi Aoyama

**Affiliations:** ^1^ Department of Radiation Oncology Niigata University Medical and Dental Hospital Niigata Japan; ^2^ Department of Radiological Technology Niigata University Graduate School of Health Sciences Niigata Japan; ^3^ Department of Radiology and Radiation Oncology Niigata University Graduate School of Medical and Dental Sciences Niigata Japan; ^4^ Department of Radiology, Division of Radiation Oncology Yamagata University Faculty of Medicine Yamagata Japan; ^5^ Section of Radiology, Department of Clinical Support Niigata University Medical and Dental Hospital Niigata Japan

**Keywords:** cone‐beam computed tomography (CBCT), fiducial marker, hypofractionated radiotherapy, image‐guided radiotherapy (IGRT), intensity‐modulated radiotherapy (IMRT), prostate cancer

## Abstract

**Purpose:**

We evaluated the setup accuracy of a three‐degree‐of‐freedom fiducial marker (3DOF‐FM)‐based setup compared to a soft tissue (ST)‐based setup in hypofractionated intensity‐modulated radiotherapy (IMRT) for prostate cancer.

**Materials and Methods:**

We analyzed the setup accuracy for 17 consecutive prostate cancer patients with three implanted FMs who underwent hypofractionated IMRT. The 3DOF‐ST‐based setup using cone‐beam computed tomography (CT) was performed after a six DOF‐bony structure (BS)‐based setup using an ExacTrac x‐ray system. The 3DOF‐FM‐based matching using the ExacTrac x‐ray system was done during the BS‐ and ST‐based setups. We determined the mean absolute differences and the correlation between the FM‐ and ST‐based translational shifts relative to the BS‐based setup position. The rotational mean shifts detected by the ExacTrac x‐ray system were also evaluated.

**Results:**

The mean differences in the anterior‐posterior (AP), superior‐inferior (SI), and left‐right (LR) dimensions were 0.69, 0.0, and 0.30 mm, respectively. The Pearson correlation coefficients for both shifts were 0.92 for AP, 0.91 for SI, and 0.68 for LR. The percentages of shift agreements within 2 mm were 85% for AP, 93% for SI, and 99% for LR. The absolute values of rotational shifts were 0.1° for AP, 0.3°, and 1.2° for LR.

**Conclusions:**

The setup accuracy of the 3DOF‐FM‐based setup has the potential to be interchangeable with a ST‐based setup. Our data are likely to be useful in clinical practice along with the popularization of the hypofractionated IMRT in prostate cancer.

## 1 INTRODUCTION

Hypofractionated radiotherapy has been in widespread use for prostate cancer treatment, based on the evidence that prostate cancer has a low α/β ratio.^1^, ^2^, ^3^, ^4^ With this radiotherapy modality, the daily patient setup using image‐guided radiation therapy (IGRT) is more important than that in conventionally fractionated radiotherapy for achieving the necessary high conformity of the dose distribution.

For the correction of a daily patient setup, there are various IGRT techniques such as kilovoltage (kV) and megavoltage (MV) portal imaging with implanted fiducial markers (FMs),^5^, ^6^, ^7^, ^8^ ultrasound,^9^, ^10^, ^11^ electronic portal imaging devices with FMs,^12^, ^13^ and kV and MV cone‐beam computed tomography (CBCT).^14^, ^15^, ^16^ In particular, the three degrees‐of‐freedom (3DOF) CBCT‐based setup has become widely used for prostate cancer, and these are the most frequently used systems in the United States.^16^ At our institution in Japan, the standard option for the verification of patients with prostate cancer is a 3DOF‐soft tissue (ST)‐based setup with CBCT, which has the potential advantage (compared to other IGRT techniques) of providing three‐dimensional volumetric images.

In contrast, an ST‐based setup with CBCT takes a longer time compared to other IGRT techniques, and as a result, its use is likely to induce increased intrafractional prostate shifts.^17^ A potential alternative to CBCT is offered by the ExacTrac x‐ray system (BrainLAB, Feldkirchen, Germany) because it has clinical advantages compared to CBCT, including a faster patient setup with six degrees‐of‐freedom (6DOF) and a reduction in image‐based radiation dose to the patient.^18^, ^19^ However, when FMs are used for prostate cancer, there is a risk that unnecessarily large shifts may be calculated because the distances among the FMs are very close compared to the size of the entire pelvis. Shi et al. reported that a rotational shift <2° may not need to be adjusted in a 6DOF‐FM‐based setup used with the ExacTrac x‐ray system.^20^ Barney and Oehler et al. investigated the accuracy of a 3DOF‐FM‐based setup using the orthogonal kilovoltage portal imaging.^7^, ^8^ However, little has been reported on the efficacy of a 3DOF‐FM‐based setup without rotational shift verification using the ExacTrac x‐ray system. We conducted this study to evaluate the accuracy of a 3DOF‐FM‐based setup with the ExacTrac x‐ray system compared to a 3DOF‐ST‐based setup with CBCT for the same prostate cancer patients who had undergone hypofractionated IMRT.

## 2 MATERIALS AND METHODS

### 2.1 Patient selection

We conducted an institutional review board (IRB)‐approved study (approval no. 2145) for prostate cancer patients in whom three FMs were implanted and who had undergone hypofractionated IMRT at our hospital between April 2014 and March 2015. A total of 17 consecutive prostate cancer patients were provided their informed consent under our IRB concerning the use of their data for research purposes.

Among the 17 patients, one patient was ineligible for this analysis because the FM implantation was not successful. Ten patients received 70 Gy in 28 fractions 4 days per week, and the other six patients received 62 Gy in 20 fractions four days per week. In total, the shift data for 400 fractions were analyzed. The patient characteristics are summarized in Table 1.

**Table 1:** Patient and treatment characteristics

Data	n = 16
Age, yrs; median (range)	72 (62–85)
Initial PSA, ng/ml; median (range)	11.2 (5.5–20.9)
Gleason score:	
≤6	2
7	10
≥8	4
Clinical stage (7th UICC):	
T1c	2
T2a	6
T2b	0
T2c	8
D'Amico risk group:	
Low	1
Intermediate	4
High	11
Radiotherapy dose (Gy):	
62 Gy in 20 fractions	6
70 Gy in 28 fractions	10

PSA, prostate specific antigen; UICC, Union for International Cancer Control.

### 2.2 Treatment planning

Three fiducial 2.0‐mm gold markers were implanted in the prostate of each patient, under rectal ultrasound guidance. Approximately 1 month after the implantation, the patient was immobilized in a VacLok™ bag (Med‐Tech, Orange City, IA, USA) in the supine position and scanned on a 16‐slice CT scanner (Lightspeed RT, General Electric Medical Systems, Waukesha, WI, USA) with a filled bladder and no special bowel preparation. Planning CT images were obtained with 1.25‐mm‐thick axial slices. The prostate, seminal vesicles (SV), fiducial markers, rectum wall, and bladder wall were contoured using the Eclipse treatment planning system ver. 8.9.17 (Varian Medical Systems, Palo Alto, CA, USA). A clinical target volume (CTV) was contoured as the sum of the prostate and the proximal one‐third of the SV. The margin of the CTV to the planning target volume (PTV) was 6 mm in the left‐right (LR), anterior, and superior directions and 5 mm in the posterior and inferior directions. A seven‐field IMRT plan was generated with 6‐MV photon beams using an anisotropic analytic algorithm and the sliding window technique.

### 2.3 Image guidance system and isocenter setup accuracy

All patients were treated on a Novalis‐Tx system (Varian), with both a Varian kV on‐board‐imager (OBI) and an ExacTrac x‐ray system. The CBCT images were obtained with the follow clinical protocol: field of view (FOV) 20 cm, matrix size 512 mm × 512 mm, slice thickness 1.0 mm, and 'full‐fan' acquisition.^17^ The ExacTrac x‐ray system images were obtained with the follow conditions: 120 kV, 160 mA, and 160 ms. The isocenter setup accuracy of each system had been ensured within 0.3 mm in each direction in a daily quality assurance protocol.

### 2.4 Patient setup

**Figure 1:**
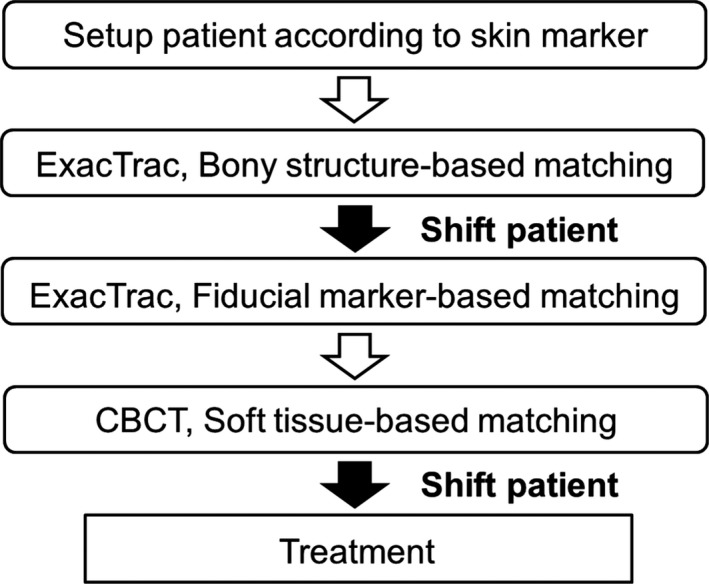
Flowchart of the study protocol.

The patient position was imaged daily in accordance with the protocol shown in Fig. 1. At treatment, the patient was initially positioned to the planning CT isocenter based on skin markers with a laser coordination system. After an initial setup, a 6DOF pelvic bony‐structure (BS) setup was carried out automatically with the use of the ExacTrac x‐ray system. Subsequently, images were obtained and FM‐based matching was performed automatically on the assumption that the setup was carried out with the use of the 3DOF‐FM‐based corrections. Pre‐treatment CBCT images were then obtained and the 3DOF‐ST‐based setup was performed manually by radiation therapists using anatomic structures in the anterior‐posterior (AP) (the prostate‐rectal interface, the anterior border of the prostate, and calcification), in the superior‐inferior (SI) (the prostate‐bladder border and calcification), and in the left‐right (LR) (the lateral borders of prostate and calcification). After the matching was checked by radiation therapists and medical physicists and approved by radiation oncologists, the radiation was delivered to the patients.

Shifts of the coordinates of the isocenter between the FM‐based and BS‐based setups and between the ST‐based and BS‐based setups were recorded in the AP axis (i.e., the positive direction corresponds to the anterior from the planning isocenter), the SI axis (the positive direction corresponds to the superior), and the LR axis (the positive direction corresponds to the left). Each rotation shift around the AP axis (yaw), the SI axis (roll), and the LR axis (pitch) detected by ExacTrac x‐ray system was also recorded.

### 2.5 Evaluation index and statistical analysis

We determined the mean absolute differences and the Pearson's correlation coefficient between the FM‐ and ST‐based translational shifts relative to the position of the BS‐based setup. We then plotted the differences between each shift against the average shift by performing a Bland‐Altman analysis to assess the fixed bias. The rotational shift in the ExacTrac x‐ray system relative to the position of the BS‐based setup was also evaluated.

## 3 RESULTS

### 3.1 Comparison of the translational shifts between the 3DOF‐FM‐based and ST‐based setups

**Figure 2:**
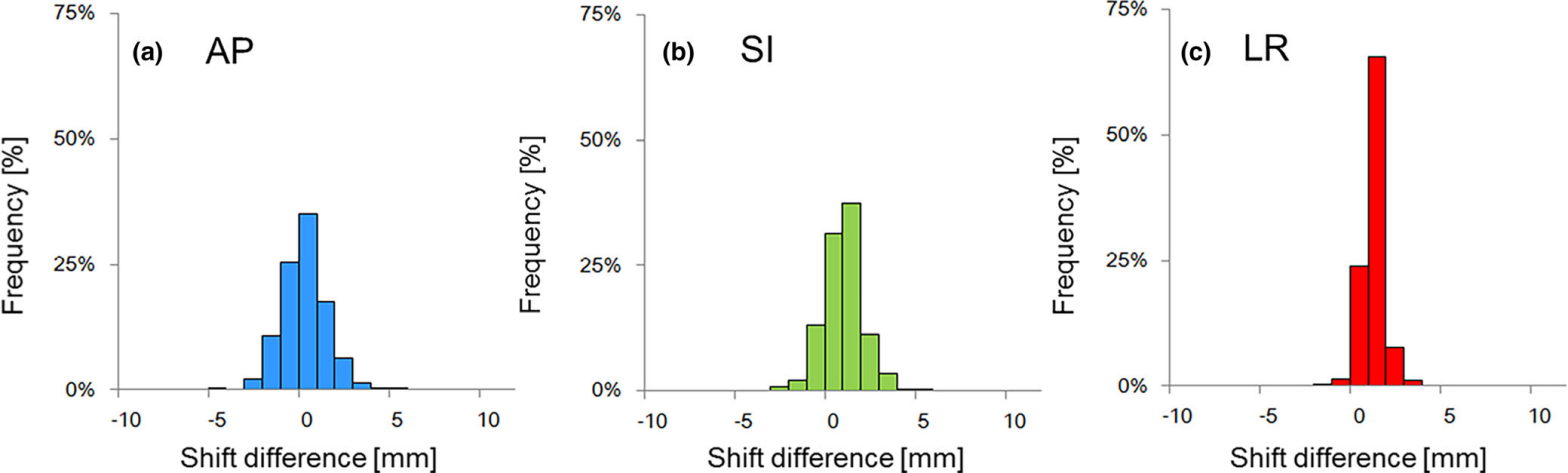
The frequency histogram for the differences between ST‐based and FM‐based translational shifts: (a) anterior‐posterior (AP), (b) superior‐inferior (SI), and (c) left‐right (LR) axes.

The mean absolute differences between the 3DOF‐FM‐based shifts and the ST‐based shifts relative to the position of the BS‐based setup for the distribution in the AP, SI, and LR axes were 0.69 mm [standard deviation (SD) 1.3 mm], 0.0 mm (SD 1.1 mm), and 0.30 mm (SD 0.58 mm), respectively (Fig. 2). The percentages of number data of the shifts in each axis in the FM‐based and ST‐based setups are shown in Table 2: 41%, 50%, and 97% of the dataset in the AP, SI, and LR axes resulted within 2 mm for both the FM‐based and ST‐based shifts, respectively. There were no data in opposed shifts over 2 mm relative to the position of the BS‐based setup.

**Table 2:** Percentage of number data of the shifts in each axis for the fiducial marker‐based setup and soft tissue‐based setup

Shifts	Soft tissue‐based setup		
	≤ −2 mm	within 2 mm	≥ 2 mm
Fiducial marker‐based setup			
AP			
≤ −2 mm	4.8%	5.0%	0%
within 2 mm	0.8%	41%	13%
≥ 2 mm	0%	3.5%	32%
SI			
≤ −2 mm	9.0%	4.0%	0%
within 2 mm	1.8%	50%	5.0%
≥ 2 mm	0%	7.5%	23%
LR			
≤ −2 mm	0%	0.3%	0%
within 2 mm	1.8%	97%	0.3%
≥ 2 mm	0%	0%	0.3%

AP, anterior‐posterior; SI, superior‐inferior; LR, left‐right.

### 3.2 Evaluation of rotational shifts of the 3DOF‐FM‐based setup

**Figure 3:**
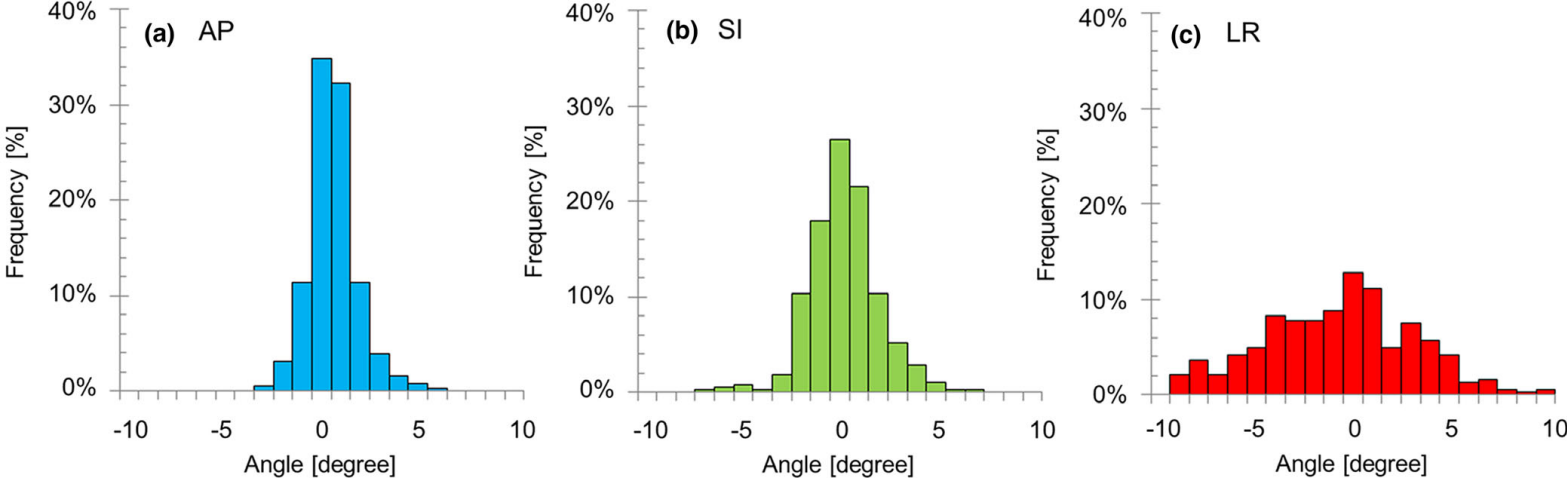
The frequency histogram for the rotational shifts detected by the ExacTrac x‐ray system after the BS‐based setup: (a) AP, (b) SI, and (c) LR axes.

The absolute values of the rotational mean shifts in the ExacTrac x‐ray system after the BS‐based setup were 0.1° (SD 1.2°) for the AP axis, 0.3° (SD 1.8°) for the SI axis, and 1.2° (SD 3.8°) for the LR axis, as shown in Fig. 3. The percentages over 3° were remarkably larger in the LR (39%) axis compared to the AP (2.6%) and SI (6.2%) axes.

### 3.3 Correlation between the 3DOF‐FM‐based and ST‐based shifts

**Figure 4:**
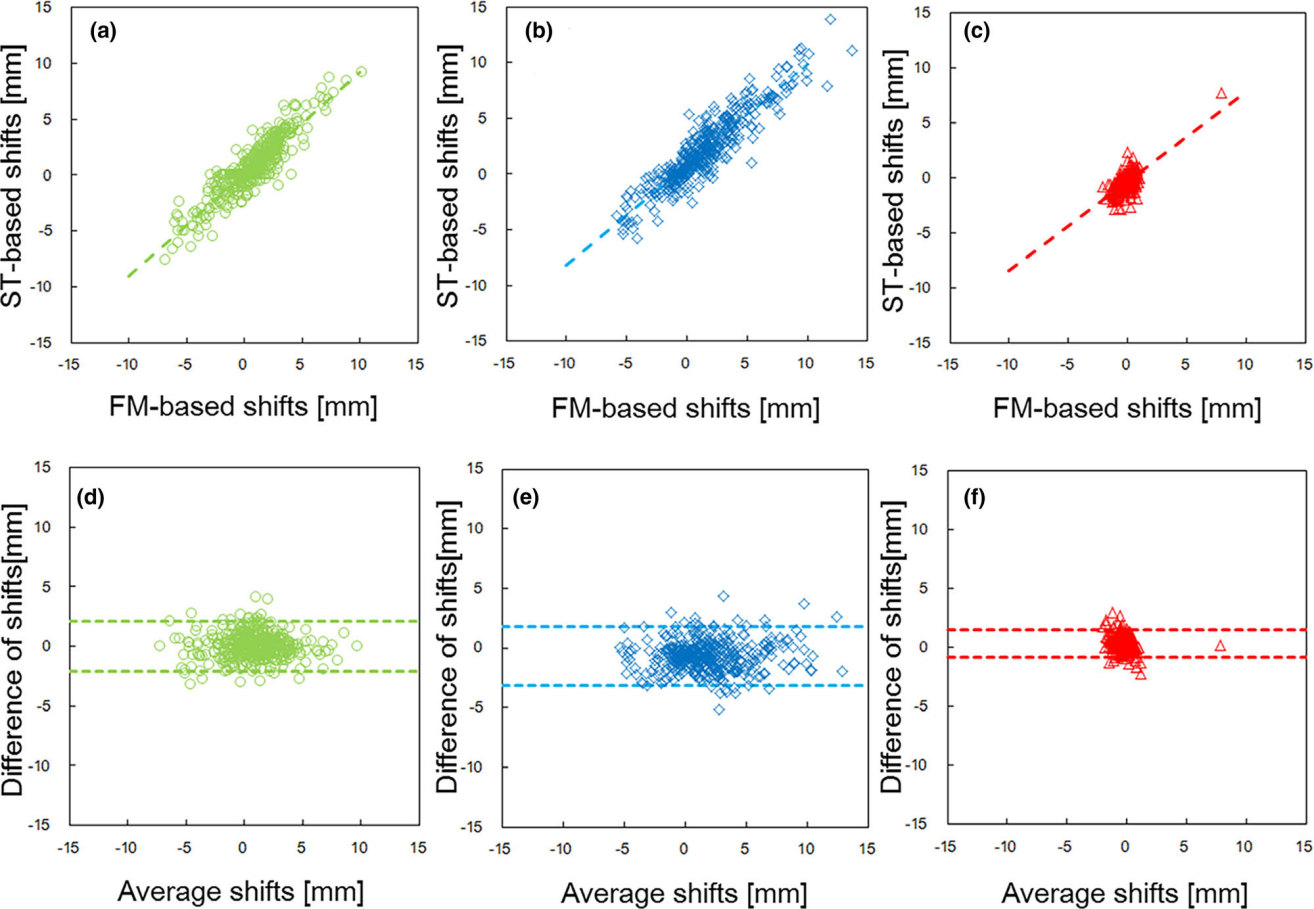
Two‐dimensional correlations (a–c) and Bland‐Altman error analysis (d–f) between the ST‐based setup and FM‐based shifts: (a) AP, (b) SI, and (c) LR axes.

The Pearson's correlation coefficients (*r*) between the 3DOF‐FM‐based and ST‐based shifts were 0.92 for the AP axis, 0.91 for the SI axis, and 0.68 for the LR axis, as shown in Fig. 4. The gradient and intercept of the dash lines which were determined using a constrained least‐square fitting were (0.90, 0.83) for the AP axis, (0.91, 0.067) for the SI axis, and (0.81, −0.30) for the LR axis. In the Bland‐Altman error analysis, the 95% confidence interval was (−3.2, 1.8) for the AP axis, (−2.1, 2.1) for the SI axis, and (−0.84, 1.4) for the LR axis, indicating no systematic bias. The percentages of shift agreements within 2 mm were 85% for the AP axis, 93% for the SI axis, and 99% for the LR axis.

## 4 DISCUSSION

To our knowledge, this is the first study to compare the 3DOF‐FM‐based setup with the ExacTrac x‐ray system and the ST‐based setup with CBCT in hypofractionated IMRT for prostate cancer. The mean absolute differences and high correlation for the isocenter shifts between the FM‐ and ST‐based translational shifts relative to the position of the BS‐based setup demonstrate that the 3DOF‐FM‐based setup has the potential to be interchangeable with the ST‐based setup.

The considerable results in shifts between the 3DOF‐FM‐based setup and the ST‐based setup in this study were consistent with those described by Barney et al.^7^ Their study compared the FM‐based setup with orthogonal kV portal imaging and an ST‐based setup with CBCT for 36 prostate cancer patients. Regarding the percentage of shift agreements within 3 mm between each setup, the same tendency was observed between Barney's study (41.3% for AP, 49.3% for SI, and 87.4% for LR) and ours (97% for AP, 99% for SI, and 100% for LR). Oehler et al. compared the setup accuracy between an FM‐based setup with orthogonal kV portal imaging and an ST‐based setup with CBCT for 20 prostate cancer patients with volumetric modulated arc therapy.^8^ They found that the shift differences were usually <2 mm in all axes. Our results also showed that the mean shift differences were <2 mm (0.69 mm for AP, 0.0 mm for SI, and 0.30 mm for LR). The shift difference in each direction may be explained by the prostate motion between ExacTrac and pre‐treatment CBCT, with larger SD in the AP and SI directions.

Several research groups have discussed the need for the verification of the rotational shifts for patient setups in prostate cancer.^18^, ^19^, ^20^, ^21^, ^22^, ^23^, ^24^, ^25^, ^26^ Shi et al. reported that the prostate IMRT with implanted FMs and the ExacTrac x‐ray system achieved <2 mm setup uncertainty in translations, and <0.25° in rotations as the overall interfractional mean error for 36 patients with prostate cancer.^20^ They also reported that a rotational shift <2° may not need to be adjusted for patient setup. Anbry et al. reported the SDs in interfraction rotation around AP, SI, and LR were 2.9°, 3.6°, and 8.0° for 18 prostate cancer patients and their results were similar to ours (1.2° for AP, 1.8° for SI, and 3.8° for LR).^26^ It is assumed that the large percentage of >3° in LR direction is caused by filling the rectum which exerts pressure on the prostate from the posterior direction. We consider the control of rectum condition are important for 3DOF‐FM‐based setup.

The results of the average rotational shifts <2° in all axes and good shift agreements with the ST‐based setup with CBCT in this study confirm the relevance of the 3DOF‐FM‐based setup using the ExacTrac x‐ray system. Considering the rotational shift verification based on the 6DOF‐FM‐based setup, there is a risk that unnecessarily large shifts may be calculated because the distance among fiducial markers are very close compared to the size of the entire pelvis. Therefore, we think that it is unwise to take the results of the rotational shift on faith, and further investigations are needed to clarify this issue. In light of the overestimation risk for the patient setup, a 6DOF‐FM‐based setup should be performed based on careful consideration.

Although the IGRT technique has been gradually developed in order to improve setup accuracy, the optimal modalities for setup in prostate cancer are not clinically well established. A 2010 survey of IGRT in the U.S. revealed that the most common disease site was genitourinary tumors in all sites for with the IGRT technique,^16^ and the survey showed that the most commonly used IGRT modalities in genitourinary tumors were volumetric‐based technologies including CBCT (55.3%). We reported that an ST‐based setup with CBCT was superior to a BS‐based setup in prostate IMRT in terms of the PTV margin.^17^ However, an ST‐based setup with CBCT has the disadvantages of a long image acquisition time, low image quality, high radiation dose to the patient, and a high cost per patient.^27^

In contrast, an FM‐based setup is an invasive method, and the matching process may have clinical issues such as marker migration and infective complication. In their recent study, Loh et al. reported that the overall rate of symptomatic infection with FM implantation was 7.7%, and eight of the patients (2.8%) required a hospital admission.^28^ Nonetheless, they recommended the FM‐based setup to allow greater accuracy of IGRT compared to ST‐based guidance with CBCT. In another report that recommended the FM‐based setup, Zelefsky et al. performed a retrospective analysis of 186 patients with prostate cancer and found that daily IGRT with FMs in combination with high‐dose IMRT was associated with an improvement in biochemical tumor control.^29^ They also observed a significant reduction in late urinary toxicity for the patients with FMs compared to the patients without FMs. Hypofractionated radiotherapy is increasingly feasible and more convenient than conventional schedules for prostate cancer patients.^3^, ^4^ Our present findings are likely to be useful in clinical practice as hypofractionated IMRT is used more commonly for prostate cancer.

This study has some limitations. First, the purpose is to compare the setup accuracy between 3DOF‐FM‐based and 3DOF‐ST‐based setups. However, the presence of FMs introduces an image artifact and could impact the 3DOF‐ST‐based setup using CBCT. As a result, the ST‐based shifts with FMs are not the exactly same as those without FMs, which may make the comparison biased. Second, the number of patients was relatively small. However, this investigation was designed as a feasibility study and we thus considered this sample size adequate to evaluate the study objective. Third, we did not evaluate the intrafractional prostate shifts. In order to determine each setup margin exactly, it may be necessary to investigate each margin by separating the two groups. However, we feel strongly that the essential results of this study are not changed regardless of these estimations.

## 5 CONCLUSIONS

We found that a 3DOF‐FM‐based setup with ExacTrac x‐ray system has the potential to be interchangeable with an ST‐based setup in hypofractionated IMRT for prostate cancer.

## CONFLICT OF INTEREST

The authors have no conflict of interest to declare.
